# Recurrence after ESD curative resection for early gastric cancer

**DOI:** 10.1186/s40792-020-01089-0

**Published:** 2021-01-06

**Authors:** Ayako Kamiya, Hitoshi Katai, Kenichi Ishizu, Takeyuki Wada, Tsutomu Hayashi, Sho Otsuki, Yukinori Yamagata, Takaki Yoshikawa, Shigeki Sekine, Tomohiko Nishi, Yuka Kawasaki, Takafumi Ito, Hideharu Domoto

**Affiliations:** 1 Department of Gastric Surgery, National Cancer Center Hospital, 5-1-1 Tsukiji, Chuo-ku, Tokyo, 104-0045 Japan; 2 Department of Pathology, National Cancer Center Hospital, 5-1-1 Tsukiji, Chuo-ku, Tokyo, 104-0045 Japan; 3 Department of Surgery, Keiyu Hospital, 3-7-3 Minatomirai, Nishi-ku, Yokohama City, Kanagawa 220-8521 Japan; 4 Department of Gastroenterology, Keiyu Hospital, 3-7-3 Minatomirai, Nishi-ku, Yokohama City, Kanagawa 220-8521 Japan; 5 Department of Diagnostic Pathology, Keiyu Hospital, 3-7-3 Minatomirai, Nishi-ku, Yokohama City, Kanagawa 220-8521 Japan

**Keywords:** Gastric cancer, Endoscopic submucosal dissection, Neoplasm metastasis, Recurrence, Gastrectomy

## Abstract

**Background:**

Endoscopic submucosal dissection (ESD) is gaining ground as a minimally invasive treatment for early gastric cancer (EGC) that has a negligible risk of lymph node metastasis. According to the 5th edition of Japanese gastric cancer treatment guidelines, annual or biannual follow-up with endoscopy is recommended, but follow-up with abdominal ultrasonography or computed tomography (CT) for surveillance of metastases is not recommended after the eCuraA resection. However, we experienced a case of lymph node recurrence following ESD resulting in eCuraA.

**Case presentation:**

A 76-year-old female received ESD for EGC in a previous hospital 4 years ago. Pathological findings were tub1, 30 mm, T1a (M), UL0, Ly0, V0, pHM-, pVM- (eCuraA) according to the 15th edition of Japanese Classification of Gastric Carcinoma. Follow-up esophagogastroduodenoscopy revealed submucosal tumor, which was suspected as a swollen lymph node by CT and endoscopic ultrasound fine-needle aspiration revealed the recurrence of gastric cancer. We performed total gastrectomy with D2 lymph node dissection. Postoperative pathological examination revealed no local recurrent tumor at the ESD site in the stomach. Swollen lymph node was diagnosed as metastasis and lymph node metastasis was limited near the cardia.

**Conclusion:**

This case provides valuable information about tumor with a minimum poorly differentiated adenocarcinoma component may develop lymph node metastasis even satisfying the guidelines criteria for curative resection.

## Background

Endoscopic submucosal dissection (ESD) is gaining ground as a minimally invasive treatment for early gastric cancer (EGC) with a negligible risk of lymph node metastasis [1]. The pathological criteria of endoscopic treatment based on retrospective examinations of surgical resection cases are feasible. However, we experienced a case of lymph node recurrence following ESD resulting in a pathologically curative resection of EGC.

## Case presentation

A 76-year-old female received ESD for EGC in the previous hospital 4 years ago. Pathological findings were tub1, 30 mm, T1a(M), UL0, Ly0, V0, pHM-, pVM- according to the 15th edition of Japanese Classification of Gastric Carcinoma [2], therefore curative resection was achieved (eCuraA). She was followed up by annual esophagogastroduodenoscopy (EGD), which indicated no evidence of metastasis in 3 years. During follow-up submucosal tumor was detected in EGD, it was suspected as a swollen lymph node in computed tomography (CT) image and endoscopic ultrasound fine-needle aspiration showed recurrence of gastric cancer. She was referred to our hospital.

There were no abnormalities in the physical examination. Laboratory studies showed within normal range. Serum CA125 level was 278 ng/mL (normal range, < 35 ng/mL), and other serum tumor markers were normal, including CEA, CA19-9 and α-fetoprotein. She received low anterior resection for rectal cancer 10 years ago, and pathological findings were tub1, T3(SS), Ly1, V1, INF b, int, pPM-, pDM-, pN0, fM0, fStage II according to the 3rd English edition of Japanese Classification of Colorectal, Appendiceal, and Anal Carcinoma. She also received partial hepatectomy for liver metastasis and upper right lobe partial excision for lung metastasis 9 years ago.

**Fig. 1:**
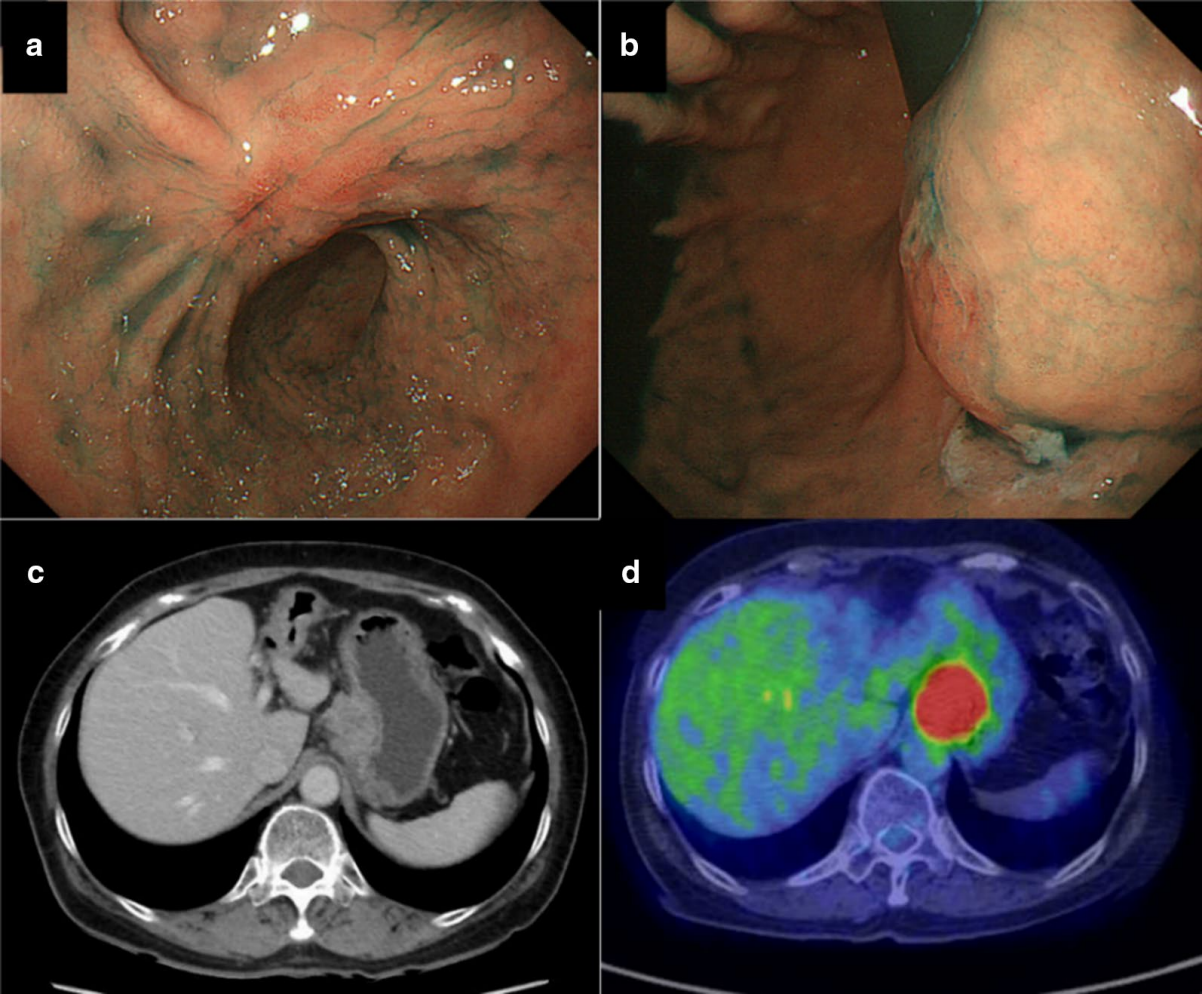
Preoperative findings. **a**, **b** EGD found ESD scar at the lesser curvature of antrum and a SMT at the lesser curvature of cardia. **c** CT shows an approximately 30-mm, high density, round mass at the lesser curvature of upper gastric body. **d** Uptake of fluorodeoxyglucose seen on PET–CT

EGD found ESD scar at the lesser curvature of antrum (Fig. 1a), and a submucosal tumor at the lesser curvature of cardia (Fig. 1b). CT showed an approximately 30-mm, high density, round mass at the lesser curvature of upper gastric body (Fig. 1c). There were neither ascites nor other evidences of metastasis. Uptake of fluorodeoxyglucose was seen on positron emission tomography–CT (Fig. 1d). Therefore, lymph node metastasis was diagnosed as resulting from the EGC originally treated by ESD.

Total gastrectomy with D2 lymph node dissection was carried out. Because tumor invaded distal part of the pancreas, distal pancreatectomy was added. The total operating time was 337 min. The intraoperative blood loss was 647 mL. Pancreatic fistula and intra-abdominal abscess (Clavien–Dindo classification: Grade 3a) occurred after surgery, and she was discharged on 74 days of operation.

**Fig. 2:**
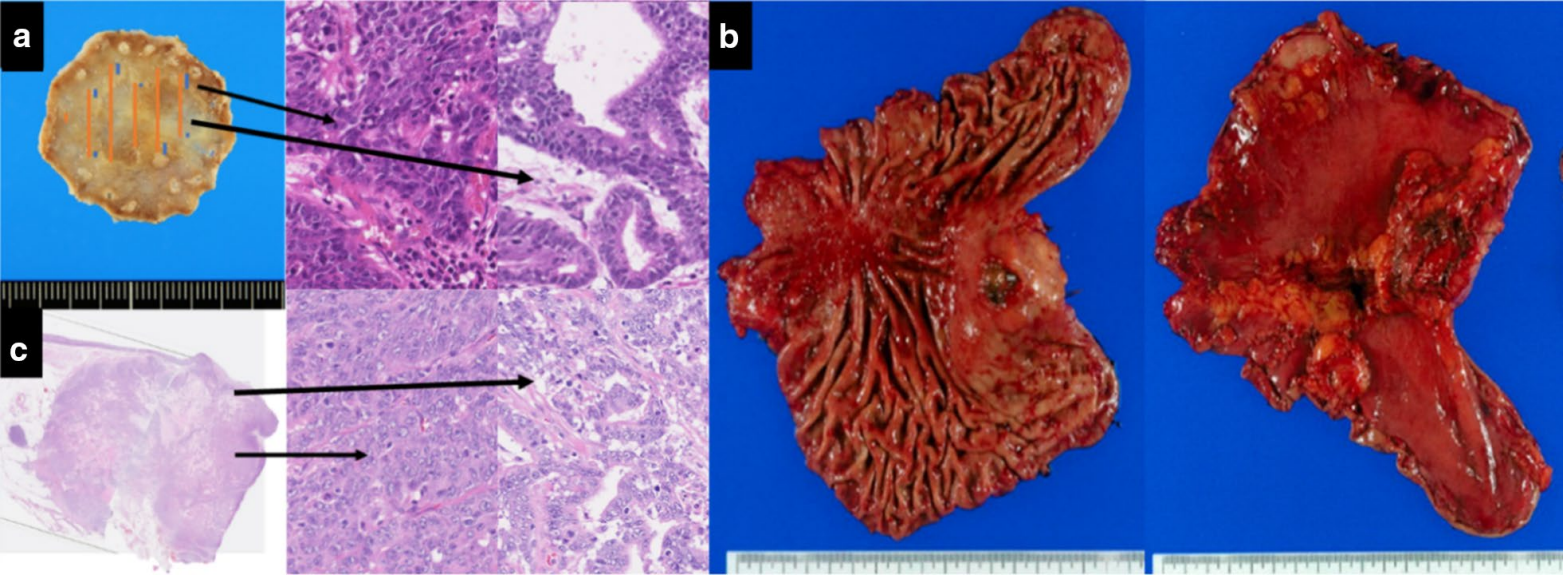
Pathological findings. **a** ESD specimen predominantly reveals a well to moderately differentiated adenocarcinoma with a poorly differentiated component which the blue lines indicate. The orange lines indicate the cancer. **b** The tumor measures 37 × 33 mm. **c** A lymph node resected by surgery reveals a moderately to poorly differentiated adenocarcinoma

The resected specimen had the submucosal tumor with ulceration on the top, which had not been observed by EGD before surgery. The tumor measured 37 × 33 mm (Fig. 2b). Postoperative pathological examination revealed no local recurrent tumor at the ESD site in the stomach. Poorly differentiated adenocarcinoma occupied the majority of the tumor and metastasis was limited to the mural tumor considered to be a lymph node near the cardia (Fig. 2c). We thought that the EGC resected by ESD was the origin of the lymph node metastasis because the swollen lymph node was a regional lymph node, there was no other primary tumor, and their pathological characters are similar. The status of HER2 expression was negative. No metastasis was observed in other lymph nodes retrieved at the surgery.

She has received adjuvant chemotherapy with S-1. No recurrence has been subsequently detected 9 months after the surgery.

## Discussion

According to the 5th edition of Japanese gastric cancer treatment guidelines, the resection is classified as endoscopic curability A (eCuraA) when all of the following conditions are fulfilled, provided the cancer is without ulcerative findings (UL0) regardless of tumor size, or with ulcerative findings (UL1) less than 3 cm in size: en bloc resection, any tumor size, histologically differentiated type-dominant, pT1a, negative horizontal margin (HM0), negative vertical margin (VM0) and no lymphovascular [3]. This case was diagnosed as eCuraA for ESD.

Gotoda et al. and Hirasawa et al. reported that no surgical EGC cases without lymphovascular involvement had lymph node metastases in intramucosal differentiated type cancers over 2 cm in size without ulceration, intramucosal differentiated type cancers less than 3 cm in size with ulceration [4, 5]. JCOG0607 included 470 patients with clinically diagnosed differentiated type intramucosal cancers without ulceration or less than 3 cm in size with ulceration, and 371 patents who had achieved curative resection had no recurrence [6]. Oda et al. reported no local recurrence and 1 metastasis in a multicenter study on long-term outcomes of eCuraA in 2723 patients with EGC [7]. This case was diagnosed as eCuraA. Follow-up with annual or biannual endoscopy is recommended after the eCuraA resection. However, follow-up with abdominal ultrasonography or CT for surveillance of metastases is not recommended after the eCuraA resection.

On this favorable trend of ESD for EGC, we experienced a case of lymph node recurrence after curative ESD for intramucosal gastric cancer. It is a rare case for we searched the core database Pubmed up to May, 2020 for the literature about the similar case using “ESD” and “eCuraA” as keywords, we found only 2 previous case reports which reported a recurrence after the eCuraA ESD resection including references in the report (Table 1) [8, 9].

**Table 1:** Recurrences after the eCuraA ESD resection

Case	Author/year	Age/sex	Differentiation	Size (mm)	UL	Recurrence	Period until recurrence
1	Hanaoka/2009 [8]	60/male	Differentiated type-dominant	55	−	Lymph node Liver	14 months
2	Fujii/2015 [9]	70/male	Differentiated type	22	+	Lymph node	17 months
3	Our case/2020	76 /female	Differentiated type-dominant	30	−	Lymph node	4 years

Only 2 previous case reports were reported a recurrence after the eCuraA ESD resection

Hanaoka et al. reported a case of nodal recurrence and liver metastasis after curative ESD of a moderately differentiated-dominant intramucosal EGC 55 mm in size, intermingled with poorly differentiated adenocarcinoma in some regions [7]. Fujii et al. reported a case of lymph node metastasis after curative ESD of a moderately differentiated intramucosal EGC, 22 mm in size accompanied by an ulcer [9].

The resected specimen revealed predominantly a well-differentiated adenocarcinoma. However, a poorly differentiated adenocarcinoma component was also identified (Fig. 2a). This component might be a cause of lymph node metastasis because the resected tumor was occupied by poorly differentiated adenocarcinoma component. Lee et al. reported that lymph node metastasis rate with or without the presence of undifferentiated type histology within differentiated type tumors was 5.1% versus 0.5%, respectively [10]. The presence of undifferentiated type histology within differentiated type tumors was found to be associated with lymph node metastasis in the multivariate analyses [10]. Although the guidelines described an undifferentiated component of the lesion exceeds 2 cm in length, the endoscopic curability is classified as C-2 (eCuraC-2), and minimal component of undifferentiated component might be risk factor of lymph node metastasis.

In the case of Fujii et al., there was no undifferentiated component but the ulcer in the tumor [9]. The existence of ulcer is regarded as a risk factor of nodal metastasis [11]. However, Gotoda et al. reported that no surgical EGC cases out of 488 cases had lymph node metastases in intramucosal differentiated adenocarcinoma with ulcer findings less than 3 cm in size [4].

## Conclusion

Careful follow-up is needed for patients with a poorly differentiated adenocarcinoma component, keeping in mind that metastasis may possibly occur even after curative resection based on the guideline criteria.

## Data Availability

Data sharing is not applicable to this article, since datasets were neither generated nor analyzed for the case report.

## Abbreviations

ESD: Endoscopic submucosal dissection
EGC: Early gastric cancer
CT: Computed tomography
EGD: Esophagogastroduodenoscopy
eCura: Endoscopic curability
UL: Ulcerative findings
HM: Horizontal margin
VM: Vertical margin
